# Editorial: Perinatal substance use and maternal mental health

**DOI:** 10.3389/fpsyt.2026.1832959

**Published:** 2026-05-11

**Authors:** Deepthi S. Varma, Amie J. Goodin, Kalyn Marie Renbarger, Catalina Lopez-Quintero

**Affiliations:** 1 Department of Epidemiology, College of Public Health and Health Professions, College of Medicine, University of Florida, Gainesville, FL, United States; 2 Pharmaceutical Outcomes and Policy, Center for Drug Evaluation and Safety (CoDES) Consortium for Medical Marijuana Clinical Outcomes Research, University of Florida, Gainesville, FL, United States; 3 School of Nursing, Purdue University, West Lafayette, IN, United States

**Keywords:** cannabis (marijuana), pregnancy care, pregnant and postpartum women, prevention interventions, research participation

Perinatal substance use is a persistent and rising concern throughout the United States. Coupled with elevated mental health risks in the postpartum period, it drives maternal morbidity and mortality (1, 2), and leads to neonatal complications, including short- and long-term cognitive, behavioral, and health problems, for the offspring 2–4). Although the relationship between substance use and maternal mental health is well documented, integrated screening and treatment services for pregnant and postpartum women remain fragmented or inaccessible to many. The Research Topic titled, “Perinatal Substance use and Maternal Mental Health,” feature eight original studies that highlight the complexity, heterogeneity in potential research or clinical interventions, and assessment of clinical intervention responses to these intersecting conditions. Collectively, this work, illustrates the prevalence and correlates of substance use during pregnancy and the postpartum period and highlights the barriers to accessing adequate perinatal care and substance use treatment or participate in research, and showcases the design of innovative interventions aimed at reducing use or improving recovery outcomes.

A core theme across the Research Topic is the nuanced understanding of substance use behaviors and motivations during pregnancy and postpartum. Perinatal substance use does not typically occur in isolation and is shaped by prior life experiences. Two papers in this Research Topic highlight the **intersections of early life adversity, resilience, and perinatal substance use**. Roland et al. found adverse childhood experiences or trauma strongly influence perinatal substance use patterns, underscoring the need for a trauma informed approach when working with pregnant and postpartum women with substance use disorders. Relatedly, Jones et al. pregnant women with low resilience already exhibit high cannabis use, rendering additional adverse childhood experiences (ACEs) only marginally influential, while those with high resilience are not protected when facing severe ACE exposure (6–10 events). These studies show that it is critical to address perinatal substance use and co-occurring mental health disorders within the context of a life course trajectory, rather than just as behaviors during pregnancy.

Another compelling contribution in this Research Topic by Vandewalle et al., highlights perinatal period as a “window of opportunity” as this research provides the health care provider’s perspective on how various sectors such as healthcare, child welfare, substance use, and child protection services need to work collaboratively to overcome systemic challenges to provide higher quality, and better integrated, care. As a direct evaluation on an application of this concept, another study in this Research Topic by Osweiler et al. designs and tests a mobile application to reduce self-stigma among women with opioid use disorder during pregnancy and postpartum, which illustrates how inclusive, technology-enabled approaches can support engagement and potentially enhance access to nonpunitive care. Physical activity-based intervention, as presented in another study within this Research Topic, Battle et al., also presented a promising angle for integrating a more holistic approach to perinatal mental and behavioral health care. Further, the study by Blair et al. examines why pregnant individuals continue or stop using cannabis and how motivations influence ongoing use. It found that cannabis use during pregnancy is common and often frequent, with multiple motivations for use strongly associated with continued use despite attempts to quit, highlighting the need for targeted clinical counseling and public health interventions.

Finally, this Research Topic contains two studies, (Varma et al.; Sojin et al.) that explored the willingness and recruitment of feasibility of marijuana exposed women, and their children, for participation in longitudinal research studies. Both studies emphasized the readiness of many women to engage in long-term research studies with their infants thereby reinforcing the feasibility of cohort studies that can help understand long-term developmental outcomes of perinatal substance use.

Taken together, these papers reinforce that the limitations in fragmented care for mental health and substance use appear to be magnified in the perinatal period. However, promising interventions are presented within this Research Topic that seek to reduce barriers associated with stigma and with limited care accessibility. Importantly, we learn from these studies that women in the perinatal period see themselves as having an active role to play in directing their mental health and substance use-related care. Women in the perinatal period report a strong willingness to engage with research and with clinical intervention in several of the studies contained within this Research Topic.

## Conclusion

Future research must first eliminate the research and clinical barriers that have historically excluded women with comorbid perinatal substance-use and mental-health disorders from studies and care. Interventions targeting trauma experiences and resilience-building skills may prove useful. Similarly, promising digital-health interventions that address self-stigma, along with holistic strategies incorporating physical activity, could effectively reduce substance use and enhance recovery outcomes.
